# *Candida albicans* and *Pseudomonas aeruginosa* Interaction, with Focus on the Role of Eicosanoids

**DOI:** 10.3389/fphys.2016.00064

**Published:** 2016-02-26

**Authors:** Ruan Fourie, Ruan Ells, Chantel W. Swart, Olihile M. Sebolai, Jacobus Albertyn, Carolina H. Pohl

**Affiliations:** ^1^Pathogenic Yeast Research Group, Department of Microbial, Biochemical and Food Biotechnology, University of the Free StateBloemfontein, South Africa; ^2^National Control Laboratory, University of the Free StateBloemfontein, South Africa

**Keywords:** *Candida albicans*, co-infection, eicosanoid, interaction, prostaglandin, *Pseudomonas aeruginosa*

## Abstract

*Candida albicans* is commonly found in mixed infections with *Pseudomonas aeruginosa*, especially in the lungs of cystic fibrosis (CF) patients. Both of these opportunistic pathogens are able to form resistant biofilms and frequently infect immunocompromised individuals. The interaction between these two pathogens, which includes physical interaction as well as secreted factors, is mainly antagonistic. In addition, research suggests considerable interaction with their host, especially with immunomodulatory lipid mediators, termed eicosanoids. *Candida albicans* and *Pseudomonas aeruginosa* are both able to utilize arachidonic acid (AA), liberated from the host cells during infection, to form eicosanoids. The production of these eicosanoids, such as Prostaglandin E_2_, by the host and the pathogens may affect the dynamics of polymicrobial infection and the outcome of infections. It is of considerable importance to elucidate the role of host-produced, as well as pathogen-produced eicosanoids in polymicrobial infection. This review will focus on *in vitro* as well as *in vivo* interaction between *C. albicans* and *P. aeruginosa*, paying special attention to the role of eicosanoids in the cross-talk between host and the pathogens.

## Introduction

Recently it has become increasingly evident that microorganisms are not only found as free floating cells, but exist as surface associated, structured and cooperative consortia, called biofilms (Douglas, 2003; Hentzer et al., 2003; Burmølle et al., 2006; Harriott and Noverr, 2011). In addition, these communities are embedded in an extracellular matrix of self-produced polymeric material. In these interactive organizations for microorganisms secreted factors and physical proximity enable metabolic interactions (Diaz et al., 2014). This often involves interkingdom interactions necessary for ecological balance and survival of certain species (Rinzan, 2009).

*Pseudomonas aeruginosa* is a Gram-negative, aerobic rod colonizing a remarkable assortment of niches, including aquatic environments, terrestrial environments and eukaryotic organisms (Pier, 1985; Tan et al., 1999). It is an opportunistic pathogen, frequently isolated from healthy humans as part of the human microbiota and is commonly found in mixed infections with the yeast, *Candida albicans* (Kaleli et al., 2006). *Candida albicans* is found as part of the normal microbiota of the skin, gastrointestinal tract and female genital tract (Morales and Hogan, 2010) and is a major cause of opportunistic infections ranging from superficial to fatal systemic infections (Sandven, 2000). Fungal infections have become increasingly troublesome in the past decades, especially in immunocompromised patients and in the hospital setting, with *C. albicans* being the most frequently isolated fungal pathogen and the most commonly isolated bloodstream pathogen (Rinzan, 2009). Selective pressure of nutrient limitation and competition between bacteria and fungi regulate the colonization of potential pathogenic microorganisms such as *C. albicans* and *P. aeruginosa*, with a disruption in this equilibrium resulting in infection by opportunistic pathogens (Calderone and Fonzi, 2001).

These two microorganisms have tendencies to form polymicrobial biofilms and as such play extensive roles in nosocomial infections, infection in immunocompromised individuals and especially in cystic fibrosis (CF) patients (El-Azizi et al., 2004; Bianchi et al., 2008; McAlester et al., 2008). This review, therefore, aims to evaluate the complex cross-kingdom relationship of these two pathogens and the impressive interaction and communication between them as well as the collateral damage to hosts caught in the cross-fire. Additionally, special attention will be given to the known immunomodulatory lipids produced by both of these microorganisms as well as the host and the role this may play during infection.

## Pathogenesis of *Pseudomonas aeruginosa*

*Pseudomonas aeruginosa* possesses numerous virulence factors including exotoxin A, proteases and lipases, released by a type II secretion system (Xcp regulon), as well as exotoxins exoS, T, U, and Y, secreted into host cells by a type III secretion system (Hogardt et al., 2004). Interestingly, it was found that *P. aeruginosa* possesses two type II secretory pathways, previously not seen in one organism (Ball et al., 2002). Additionally, pyoverdine, rhamnolipids, lipopolysaccharide (LPS) and pili also form part of this formidable pathogen's virulence arsenal (Gilligan, 1991; Méar et al., 2013). A study by Bianchi et al. (2008) showed that *P. aeruginosa* impairs the engulfment of apoptotic cells through the action of yet another virulence factor, the phenazine, pyocyanin (PYO) (Gibson et al., 2009). Interestingly, it has been shown that multiple drug resistant strains of *P. aeruginosa* show decreased production of PYO, and thus have a reduction in virulence (Fuse et al., 2013). As previously mentioned, *P. aeruginosa* forms biofilms, and a universal model for the formation of *P. aeruginosa* biofilm formation was suggested (O'Toole et al., 2000; Klausen et al., 2003). According to this model, *P. aeruginosa* cells move by means of flagella to an adequate surface and movement along this surface is accomplished through type IV-pili. Cells aggregate, leading to microcolony formation. During maturation, large mushroom-shaped structures are formed. Klausen et al. (2003) proposed an alternate model, with evidence indicating that flagella do not play a role in the attachment of *P. aeruginosa* cells. The formation of *P. aeruginosa* biofilms are, however, highly dependent on the carbon source. Additionally, the circumstances during growth, such as flow vs. stationary growth, might elicit large morphological changes.

In addition to the previously mentioned factors, the resistance of *P. aeruginosa* to antimicrobial agents is key to its pathogenic capabilities. Various mechanisms for antibiotic resistance in *P. aeruginosa* biofilms have been proposed (Drenkard, 2003). These include the reduced transport of antimicrobial agents in the biofilm due to extracellular matrix and accompanied nutrient and oxygen limitation of cells deeply embedded in the biofilm. This causes a decrease in metabolic activity of the cells. Antibiotic resistant persisters embedded in the biofilm structure, stress responses of the cells, efflux pumps and quorum sensing among cells may all contribute to the increased resistance observed in bacterial biofilms. Evidence also suggest that a protein, PvrR, regulates susceptibility and resistance phenotypes of *P. aeruginosa* (Drenkard and Ausubel, 2002; Benamara et al., 2011). An impact on lipid composition is also speculated due to differential protein expression in biofilms. In this regard, Benamara et al. (2011) examined the effect of biofilm formation on inner membrane lipid composition in *P. aeruginosa* that indicated a reduced amount of uneven numbered phospholipids. In addition, an increase in long chain phosphatidylethanolamines was observed, suggesting an increase in bilayer lipid stability and a decrease in membrane fluidity.

## Pathogenesis of *Candida albicans*

*Candida albicans* is a dimorphic yeast, meaning that both yeast and hyphal morphology is shown, with a tendency to form drug resistant biofilms (Ramage et al., 2001). The ability of this microorganism to switch between the planktonic single yeast cell and hyphal morphologies has a major influence on its virulence (Andes et al., 2004; Pierce, 2005; Bruzual et al., 2007; Brand et al., 2008; Gil-Bona et al., 2015). In addition to this morphological plasticity, the aggressiveness of *C. albicans* colonization is due to a collection of other virulence factors. These include adhesins (biomolecules that enable binding to host cells or host cell ligands), lipolytic and proteolytic enzymes and phenotypic switching (white to opaque switching) (Calderone and Fonzi, 2001). Interaction of *C. albicans* with the host is largely accomplished by contact with the *C. albicans* cell surface and subsequent biofilm formation (Gow and Hube, 2012). Ramage et al. (2001) investigated the formation of *C. albicans* biofilms through visualization of the biofilms at various stages of development and monitoring the metabolic activity of the cells. Through scanning electron microscopy (SEM) and confocal scanning laser microscopy (CSLM) a dense network of hyphae and yeast cells in a matrix of exopolymeric material was visualized in the matured biofilm. This study also tested the effect of antifungal drugs on *Candida* biofilms and planktonic *C. albicans* cells. It was found that cells in the biofilm had a 250-fold increase in resistance against fluconazole. Dumitru et al. (2004) argued that this increased resistance might be due to the hypoxic conditions found in biofilms, with anaerobically grown *C. albicans* also showing resistance against certain antifungal agents. Interestingly, a study by Kuhn et al. (2002) evaluated a range of antifungal agents against *Candida* and found that sub-inhibitory concentrations of certain antifungals elicited alterations in biofilm formation.

## Interaction between *Pseudomonas aeruginosa* and *Candida albicans In vitro*

### Physical/direct interaction

**Figure 1:**
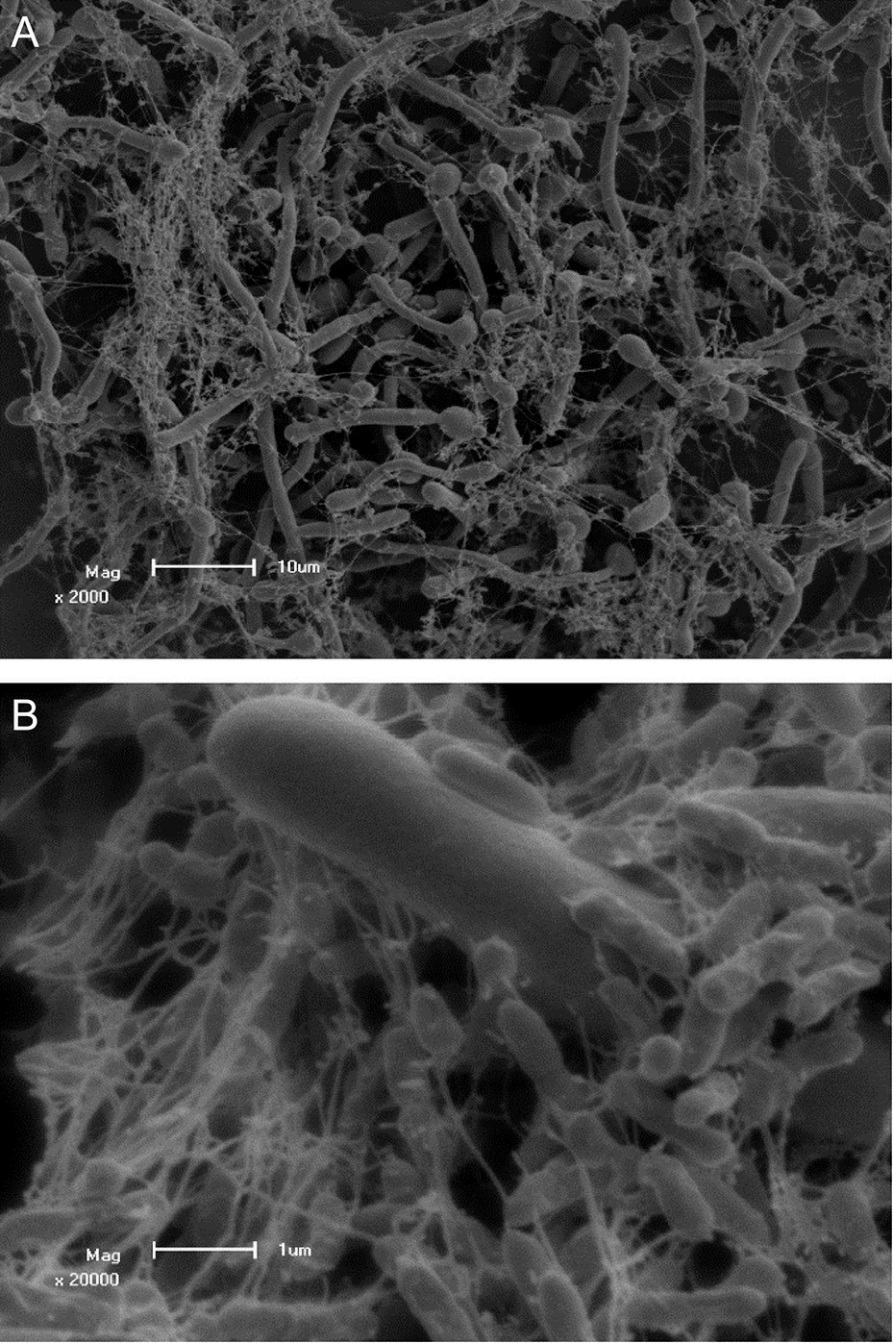
**Scanning electron micrographs of *Candida albicans* colonized by *Pseudomonas aeruginosa* PAO1 showing adhesion to *C. albicans* hyphae**. Scale bars represent **(A)** 10 μm and **(B)** 1 μm. Co-culture of *C. albicans* and *P. aeruginosa* was performed and visualized by Ruan Fourie.

Several studies were conducted to evaluate the interaction of *P. aeruginosa* and *C. albicans*. The antagonistic interaction of *C. albicans* and *P. aeruginosa* was examined by Brand et al. (2008) who showed that *P. aeruginosa* cells kill *C. albicans* hyphal cells, but not *C. albicans* yeast cells. The deadly effect on *C. albicans* is thought to be due to PYO, which alters the cell wall of *C. albicans* (Kerr et al., 1999). Further research into this interaction provided evidence that there is a difference in *P. aeruginosa* mediated *C. albicans* killing among different morphotypes of *C. albicans* (Rinzan, 2009). Increased susceptibility to the killing effect of *P. aeruginosa* was seen with filamentous cells compared to planktonic counterparts, similar to the study by Brand et al. (2008), as well as a reversion of germ tube formation in the presence of *P. aeruginosa*. Further analysis of this interaction indicated that attachment of *P. aeruginosa* and *C. albicans* killing is mediated by lectin-carbohydrate interaction, type IV pili as well as mannans. The authors also speculated on the possible involvement of O-linked mannans in the survival of *C. albicans* yeast cells during combined incubation, as was proposed previously (Brand et al., 2008; Rinzan, 2009). In Figure 1, scanning electron micrographs of a dual species biofilm with *C. albicans* and *P. aeruginosa* is seen showing extensive colonization of *C. albicans* cells by *P. aeruginosa*.

### Indirect interaction

#### Role of *Pseudomonas aeruginosa* quorum sensing molecules during *In vitro* interaction

The interaction of *C. albicans* and *P. aeruginosa* is mediated by quorum sensing molecules (QSM), produced by both organisms (Cugini et al., 2007). The bulk of Gram-negative bacterial quorum sensing systems utilize *N*-acyl homoserine lactones (AHL) that bind and activate their respective transcriptional activators (R protein) to induce expression of target genes (de Kievit and Iglewski, 2000). When adequate population density is reached by bacterial cells, AHL concentrations are high enough to induce these transcriptional changes.

**Figure 2:**
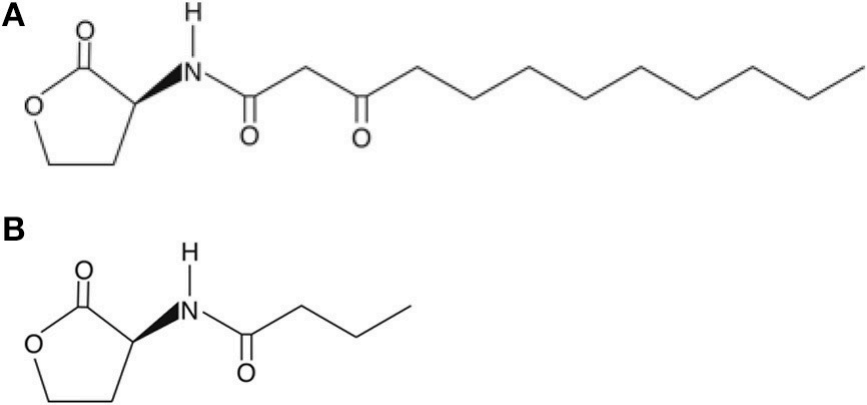
**Structures of (A) 3-oxododecanoyl-L-homoserine lactone at the top and (B) butanoylhomoserine lactone underneath**.

Two AHL-dependent QS systems were identified in *P. aeruginosa*, namely the *las* and *rhl* systems (de Kievit and Iglewski, 2000). 3-oxododecanoyl-L-homoserine lactone (3-oxo-HSL) is an autoinducer with its production directed by LasI autoinducer synthase (*las* QS system). The production of another autoinducer, butanoylhomoserine lactone, is similarly regulated by RhlI autoinducer synthase (*rhl* QS system). These bind and activate their respective transcriptional activators LasR and RhlR (Passador et al., 1993; Pearson et al., 1995). These QSMs may regulate up to 10% of the genome of *P. aeruginosa* depending on culture conditions (Hentzer et al., 2003; Wagner et al., 2003). The structures of these autoinducers are shown in Figure 2.

The QSM, 3-oxo-HSL, was studied for its effect on cell adherence in polymicrobial biofilms of *P. aeruginosa* and *C. albicans* (Ovchinnikova et al., 2012). The study showed that mutant *P. aeruginosa* strains lacking the *lasI* gene for the LasI autoinducer synthase was unable to adhere to *C. albicans* hyphae, while a *P. aeruginosa* strain without the mutation was able to adhere to *C. albicans* cells. The study suggested that 3-oxo-HSL is needed for the adherence of *P. aeruginosa* cells to *C. albicans* hyphae, because 3-oxo-HSL is needed for the production of surface adherence proteins on *P. aeruginosa* cells. A study by McAlester et al. (2008) showed that if cell free supernatant from high 3-oxo-HSL producing *P. aeruginosa* strains is added to *C. albicans* cultures, the yeast to hyphal switch is inhibited. *Pseudomonas aeruginosa* strains that produced low amounts of 3-oxo-HSL did not inhibit the yeast to hyphal switch when the supernatants of their cultures were added to *C. albicans* cultures, suggesting that 3-oxo-HSL affects yeast morphology in a dose dependent manner. To ensure that the 3-oxo-HSL was the cause of the inhibition of morphological switch, pure 3-oxo-HSL was added to a *C. albicans* culture with the same results obtained. The reaction of *C. albicans* toward 3-oxo-HSL may lead to the dispersal of *C. albicans* cells in the presence of *P. aeruginosa* (Morales and Hogan, 2010; Ovchinnikova et al., 2012). These studies thus show that AHLs are not only important for bacterial communication, but are responsible for considerable interaction with other microorganisms such as *C. albicans*.

In addition to AHLs, a QS signal, 2-heptyl-3-hydroxyl-4-quinolone signal or *Pseudomonas* quinolone signal (PQS), was later identified and this molecule is released in the late exponential phase (Pesci et al., 1999; Lépine et al., 2003). The production of PQS can be induced by the LasI/R system and inhibited by the RhlI/R system (De Sordi and Mühlschlegel, 2009). Strikingly, PQS was shown to have both a damaging effect on *P. aeruginosa* through a pro-oxidative effect, as well as an anti-inflammatory effect (Haussler and Becker, 2008). The authors speculate that this contradictory effect drives survival of the fittest through selection of phenotypic variants able to survive in stressful conditions and molding populations sufficiently adapted. It also modulates swarming motility of *P. aeruginosa* (Déziel et al., 2004; Ha et al., 2011). In addition to PQS, its immediate precursor, 2-heptyl-4-quinolone (HHQ), has been shown to repress *C. albicans* biofilm formation (Reen et al., 2011).

**Figure 3:**
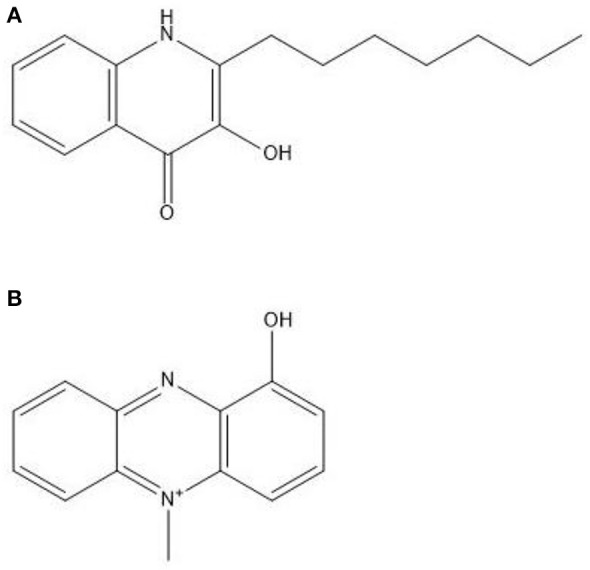
**Structures of (A) *Pseudomonas* quinolone signal at the top and (B) pyocyanin underneath**.

PQS (Figure 3A) induces the formation of several virulence factors, including phenazine compounds (Phelan et al., 2014). Of these, PYO or methyl-1-hydroxyphenazine (Figure 3B), is the best studied. Pyocyanin is a chloroform soluble compound with a blue color (Cox, 1986). It has been shown to be a QSM produced in the early stationary phase (Hernandez et al., 2004; Price-Whelan et al., 2007) and a study by Dietrich et al. (2006) suggested that PYO is a terminal signaling molecule that controls its own cycling. It plays a major role in maintaining NADH/NAD^+^ ratio stability in *P. aeruginosa* cells when they encounter oxygen limiting conditions due to the limited fermentation capability of *Pseudomonas* (Dietrich et al., 2006). Pyocyanin can then act as an alternate terminal electron acceptor and decrease the NADH/NAD^+^ ratio in the stationary phase of growth. It can then later be reoxidized by oxygen when it becomes available and this could be a mechanism for the production of reactive oxygen species (ROS). Phenazines, including PYO, phenazine-1-carboxylic acid, 1-hydroxyphenazine and phenazine-1-carboxamide play extensive roles in the interaction between *Pseudomonas* species and eukaryotes, including fungal microorganisms (Kaleli et al., 2006; Phelan et al., 2014).

In addition, PYO has antimicrobial activity against a wide range of cells including a bactericidal effect against a wide variety of bacterial species with Gram-positive species being more susceptible than Gram-negative species (Hassan and Fridovich, 1980). Interestingly, *Pseudomonas* species seem to be resistant to this bactericidal effect (Baron and Rowe, 1981). It is also toxic to eukaryotic cells (O'Malley et al., 2003). The mechanism of this effect is due to the ability of this compound to undergo non-enzymatic redox cycling intracellularly, resulting in the generation of ROS (Gloyne et al., 2011). Another effect of PYO is the reduction in cyclic adenosine monophosphate (cAMP) (Kerr et al., 1999). This enables PYO to inhibit the shift from yeast to hyphal morphology in *C. albicans* because the yeast-mycelium transition is promoted by increased levels of intracellular cAMP. Gibson et al. (2009) observed a red pigment with the co-incubation of *P. aeruginosa* and *C. albicans* produced during close proximity of the yeast and bacterial cells. This pigment was localized in fungal cells. The authors speculate that *C. albicans* enzymes participate in the formation of this product intracellularly. The precursor of this red pigment was identified as 5-methylphenazine-1-carboxylic acid (5-MPCA) through the use of *P. aeruginosa* strains with disruptions in the phenazine biosynthesis pathway. The presence of the red pigmented compound was linked to significant repression of *C. albicans* viability. In addition, a recent study indicated that the phenazines, phenazine-1-ol, phenazine-1-carboxylic acid and phenazine-1-carboxamide, has a synergistic effect with three antifungals: fluconazole, itraconazole and clotrimazole against *Candida* species (Kumar et al., 2014). This then suggests that the presence of phenazine producing organisms such as *Pseudomonas* can drastically alter the treatment of simultaneous fungal infection.

#### The role of *Candida albicans* quorum sensing molecules during *In vitro* interaction

**Figure 4:**
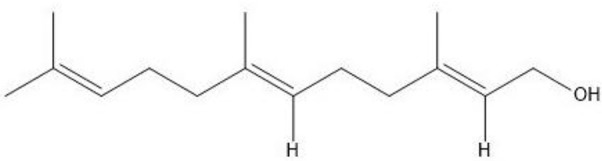
**Structure of farnesol**.

*Candida albicans* has also been shown to produce QSMs (Hornby et al., 2001). The QSM, farnesol (Figure 4), inhibits germ tube formation and also caused a morphological shift from predominant mycelial state to predominant yeast morphology, indicating the effect as 3-oxo-HSL. The effect on the morphology of *C. albicans* is thought to be due to inhibition of the *Ras1*-controlled pathway involved in hyphal growth (Morales and Hogan, 2010). Recently, farnesol was identified to attract macrophages in hosts (Hargarten et al., 2015). The authors speculate that engulfment and movement of these immune cells then aid in dissemination as macrophages are killed by *C. albicans* after engulfment. Farnesol induces the generation of ROS which could play a role in the competition of *C. albicans* with bacteria. Resistance of *C. albicans* to oxidative stress has also been shown to be linked, in part, to farnesol (Westwater et al., 2005). A study by Cugini et al. (2007) indicated that farnesol inhibits *P. aeruginosa* PQS and a subsequent virulence factor, PYO, whose production is controlled by PQS, in a dose dependent manner. Interestingly, there was no effect on the overall growth of *P. aeruginosa*. Additionally, when *P. aeruginosa* was co-cultured with *C. albicans*, reduction in PQS and PYO produced by *P. aeruginosa* was also observed, suggesting that high enough concentrations of farnesol is produced by *C. albicans* to exert an effect on *P. aeruginosa*. Later, the same research group found that *C. albicans* and its secreted factors increase PQS and butanoylhomoserine lactone in *lasR* defective mutants of *P. aeruginosa*, with a downstream increase in phenazine production (Cugini et al., 2010). The authors speculated that oxidative stress may trigger downstream quorum sensing pathways.

As seen with proteomic analysis by Jones-Dozier (2008), a possible decrease in virulence of *P. aeruginosa* is evident when exposed to farnesol, due to alterations in protein expression of *P. aeruginosa* subjected to this compound. In addition to decreasing PQS and PYO production, farnesol inhibits swarming motility in *P. aeruginosa* (McAlester et al., 2008). Rhamnolipids, a class of glycolipids, play a role in swarming motility and has been implicated as playing part in the development of ventilator associated pneumonia (VAP) (Köhler et al., 2010). Due to the fact that rhamnolipid production is partly regulated by PQS, the mechanism of decreased swarming motility may be due to the reduction in PQS production by farnesol.

#### Other factors influencing *In vitro* interaction

##### Iron availability

In a study by Purschke et al. (2012), *C. albicans* exhibited a lower metabolic activity in mixed biofilms with *P. aeruginosa* when compared to single species biofilms. Secretome analysis of the proteins of the single vs. mixed species biofilms revealed an overall increase of secreted proteins of mixed species biofilms of *C. albicans* and *P. aeruginosa* compared to the single species counterparts. This increase was largely found to be due to increased secreted proteins by *P. aeruginosa*. Interestingly, a large proportion of the increased protein production was attributed to a siderophore, pyoverdine, specific to *Pseudomonas*. This increase in pyoverdine was thought to be due to the increased iron utilization by the two species in the mixed biofilm. This was confirmed by the addition of iron, which abolished the production of pyoverdine. The authors speculated sequestration of available iron by pyoverdine results in decreased availability to *C. albicans*, although *C. albicans* is able to utilize iron bound to certain other microbial siderophores. Recent evidence suggests that this phenomenon may not be of importance during *in vivo* interaction (Lopez-Medina et al., 2015). In this study, *C. albicans* secreted factors significantly reduced pyoverdine and another siderophore, pyochelin, expression by *P. aeruginosa* during gastrointestinal colonization in a murine model. This decrease of expression by *P. aeruginosa* was linked to diminished virulence of *P. aeruginosa* with growth unaffected. The authors suspect the heterogeneity of the biofilms between *in vivo* and *in vitro* studies may cause the differential results.

Recently, Trejo-Hernández et al. (2014) found that hypoxia influences the ability of *P. aeruginosa* to inhibit *C. albicans* filamentation *in vitro* compared to aerobic conditions. This was attributed to decreased AHL produced by *P. aeruginosa* in the presence of *C. albicans*. Previously, it was shown that hypoxic conditions promote filamentation in *C. albicans* and reduces farnesol production and response to it (Dumitru et al., 2004). Additionally, the authors also speculated that competition for iron may also be greater during hypoxia (Synnott et al., 2010; Trejo-Hernández et al., 2014). Therefore, both the interaction of *P. aeruginosa* with *C. albicans*, the concentration of oxygen and iron competition influences the production of AHLs (Trejo-Hernández et al., 2014). The authors also found that proteins known to play roles in iron uptake in *P. aeruginosa* through siderophores were upregulated in mixed biofilms, confirming previous observations. Additionally, iron supplementation increased the growth of *P. aeruginosa* in single and mixed biofilms, with this effect not seen with *C. albicans*. This increase in growth of the bacterium may increase the destruction of the fungal population. Lamont et al. (2002) indicated that pyoverdine may act as a signaling molecule to modulate other virulence factors including exotoxin A and pyoverdine itself. Because pyoverdine production is increased in mixed biofilms, the virulence of *P. aeruginosa* might also be upregulated in mixed biofilms (Trejo-Hernández et al., 2014). Additionally, PQS as well as products of the PQS system including rhamnolipids and PYO, were upregulated. A significant increase in *P. aeruginosa* mutability frequency was seen with a large number of antibiotic resistant mutant phenotypes arising over time. The authors speculate that the decreased catalase activity observed in mixed biofilms may result in increased oxidative stress, concomitantly increasing mutability. In the case of *C. albicans*, the same trend was seen with hypermutability arising with a high frequency of antimicrobial resistant phenotypes, possibly attributed to the increased oxidative stress caused by PYO produced by *P. aeruginosa*. Additionally, *C. albicans* iron dependant processes, including aerobic respiration, were downregulated. Glycolytic enzyme activity in *C. albicans* was also altered, with PYO possibly attributing to this and leading to other pathways for energy utilization. To confirm the increased virulence of *C. albicans* and *P. aeruginosa* in mixed biofilms, the authors used a rat infection model. *Candida albicans* was shown to promote pathogenicity of *P. aeruginosa*. Therefore, the ability of these pathogens to compete for iron may alter population dynamics and influence the nature of the interaction.

##### Bacterial cell wall components

In addition to various secreted factors produced by *P. aeruginosa* in polymicrobial interaction (Holcombe et al., 2010), bacterial LPS were shown to have adverse effects on *Candida* spp. biofilms (Bandara et al., 2010). The same group later confirmed these results by evaluating the effect of *P. aeruginosa* LPS on *C. albicans* (Bandara et al., 2013). The study suggested a decrease of *C. albicans* filamentation and biofilm metabolic activity, including glycolysis, and growth with the addition of high concentrations of *P. aeruginosa* LPS. In addition to this, peptidoglycan was shown to trigger filamentation in *C. albicans* (Xu et al., 2008).

##### Ethanol

Chen et al. (2014) evaluated the effect of *C. albicans* produced ethanol on *P. aeruginosa* and found that ethanol stimulated adhesion and biofilm formation of *P. aeruginosa*. In addition, swarming motility by *P. aeruginosa* decreased and a stimulation of phenazine derivatization and production of 5-MPCA by *P. aeruginosa* in the presence of ethanol was observed. The authors speculate that there is a positive feedback loop where *C. albicans* ethanol production increases *P. aeruginosa* 5-MPCA production and biofilm formation. In turn, 5-MPCA stimulates ethanol production in *C. albicans* (Morales et al., 2013).

##### Extracellular DNA

A recent study also identified extracellular DNA as a large factor in biofilm formation by *C. albicans* (Sapaar et al., 2014). Low amounts of extracellular DNA (1.0 μg/mL) was shown to promote biofilm formation and increase biofilm stability, whereas higher concentrations (10 μg/mL) hampered the formation of biofilms by *C. albicans* as well as the stability of the biofilms. The study also indicated that the source of the extracellular DNA, whether it is *C. albicans*, or from bacterial sources such as *P. aeruginosa*, does not matter. This increase in biofilm formation by *C. albicans* due to extracellular DNA may increase the virulence of the fungus. Evidence also suggest that the concentration of extracellular DNA can reach 4 mg/mL in CF patient sputum samples, raising the question if this facet of interaction might have clinical relevance (Sapaar et al., 2014).

**Figure 5:**
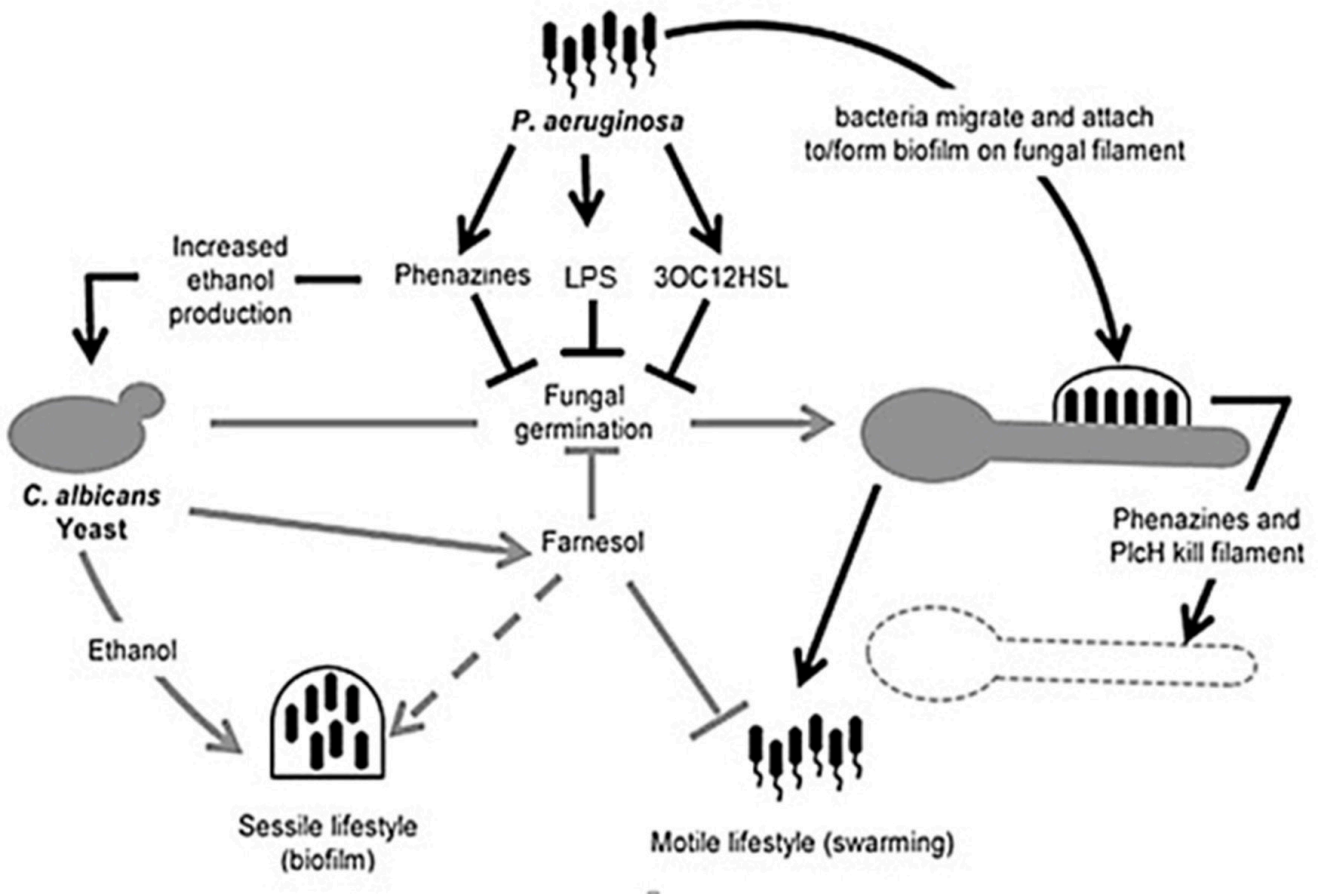
**Illustration of competition between *Candida albicans* and *Pseudomonas aeruginosa***. *Pseudomonas aeruginosa* attaches to *C. albicans* hyphae and kills hyphal cells through secreted hydrolytic enzymes such as hemolytic phospholipase C (PlcH) and phenazines such as pyocyanin and 5-methylphenazine-1-carboxylic acid (5-MPCA). 3-oxo-homoserine lactone produced by *P. aeruginosa* and phenazines inhibit filamentation by *C. albicans*, similar to farnesol, produced by *C. albicans*. *Pseudomonas aeruginosa* lipopolysaccharide (LPS) inhibits *C. albicans* filamentation. Ethanol production is increased by the fungus, inhibiting the motility of *P. aeruginosa* (adapted from Lindsay and Hogan, 2014).

A summary of several facets of interaction between *C. albicans* and *P. aeruginosa* can be seen in Figure 5.

## Interaction between *Pseudomonas aeruginosa* and *Candida albicans In vivo*

A high number of *C. albicans* nosocomial infections are polymicrobial with *P. aeruginosa* a frequent co-isolate in blood stream infections and pneumonia (Lindsay and Hogan, 2014). Kerr (1994) was the first to describe the anticandidal activity of *P. aeruginosa in vivo*. The study evaluated lung infection of three surgery patients postoperatively with inhibition of *C. albicans* growth seen after *P. aeruginosa* colonization. This inhibition was confirmed with the regrowth of *C. albicans* seen after eradication of *P. aeruginosa*, even with fluconazole treatment. Additional *in vitro* susceptibility experiments confirmed the suppression of *Candida* growth by *P. aeruginosa*. Gupta et al. (2005) evaluated 300 burn patients over 2 years and found repression of *Candida* spp. in the presence of *P. aeruginosa*. Several studies also indicate that prior colonization of *Candida* may promote susceptibility of the host to *P. aeruginosa* infection (Roux et al., 2009; Hamet et al., 2012; Xu et al., 2014). Nseir et al. (2007) reported that antifungal treatment during *Candida* spp. tracheobronchial colonization may be associated with reduced risk for *P. aeruginosa* colonization. The case is strengthened by Azoulay et al. (2006), who reported a possible link between *Candida* colonization of the respiratory tract and an increased risk for *Pseudomonas* VAP. Roux et al. (2013) reported that the Th1-Th17 immune response, associated with *Candida* colonization, caused a decrease in *P. aeruginosa* phagocytosis by alveolar macrophages, the primary innate immune response against bacterial invasion. The adaptive Th1-Th17 response evoked by *C. albicans* invasion, is characterized by an increase in interferon ɤ(IFNɤ), as well as an increase in interleukin-17 (IL-17) and decrease in IL-2. The authors suggest that the increase in IFNɤ, associated with the Th1-Th17 response, is responsible for the inhibition of bacterial phagocytosis, through inhibiting expression of scavenger receptors on alveolar macrophages.

Remarkably, contradictory results to the notion that *P. aeruginosa* infection is more aggressive after prior *C. albicans* colonization, was provided by Ader et al. (2011). In a murine model, *C. albicans* short term colonization prior to *P. aeruginosa* colonization caused a reduction in *P. aeruginosa* bacterial load compared to the absence of *C. albicans* colonization (Ader et al., 2011). Additionally, a reduction in *P. aeruginosa* induced lung injury was observed with the prior colonization of *C. albicans*. Interestingly, this effect was reversed with treatment by the antifungal caspofungin during *C. albicans* colonization. *Candida albicans* initiates alveolar innate immunity in a murine model, protecting the host against subsequent *P. aeruginosa* infection (Mear et al., 2014). The authors showed that prior *C. albicans* infection induces interleukin-17 (IL-17) and IL-22 secretion through innate lymphoid cell recruitment. The cytokines produced, induce the production of antimicrobial peptides as well as the mobilization of phagocytic cells. In a murine gut model, *C. albicans* secreted factors inhibited expression of siderophores as well as cytotoxic molecules by *P. aeruginosa*, reducing the virulence of the bacteria (Lopez-Medina et al., 2015). Due to this, increased survival of the host was observed during co-incubation of *P. aeruginosa* with *C. albicans*. Interestingly, Neely et al. (1986) demonstrated increased mortality in a murine model when *C. albicans* infection was preceded by *P. aeruginosa*. This reciprocal effect may also be due to alterations in innate immune response, as Faure et al. (2014) reported that the *P. aeruginosa* type III secretion system induced IL-18 secretion causing substantial neutrophil recruitment and host cell damage, and decreased IL-17 secretion in a mouse model, possibly leading to the reduced clearance of pathogens.

The co-infection of *P. aeruginosa* and *C. albicans* has been well documented in cystic fibrosis (CF) patients. CF is one of the most commonly encountered autosomal recessive disorders found, with the occurrence varying in race (Andersen, 1938). The disease is caused by a genetic disorder where a mutation exists in the CF transmembrane conductance regulator (CFTR) gene (Delhaes et al., 2012). CF is a disease with two pathophysiological properties, namely pancreatic insufficiency with malnutrition and also airway infections due to extremely viscous secretions (Andersen, 1938). The increased viscosity of lung secretions is thought to be due to the increased sodium absorption of the respiratory epithelium and the defective regulation of chloride ion secretion (Gilligan, 1991). This is thought to be the reason why CF patients have comparatively dehydrated surface liquid which leads to defective mucociliary clearance. The thick bronchial mucus traps viral particles, fungal spores and bacteria and provides a suitable environment for the fungal spores and bacteria to grow, causing infection (Delhaes et al., 2012). Ninety percent of deaths in CF are due to pulmonary dysfunction and in effect, chronic airway infection (Gilligan, 1991). A study by Güngör et al. (2013) evaluated the most prevalent fungal colonization in Turkish CF patients. The most prevalent fungal microorganisms isolated from these CF patients, were shown to be *C. albicans* at 62.5% (30 patients). In addition, fungal-bacterial co-colonization in the CF patients was shown to be 98.2% in these *C. albicans* infections. The most frequent bacterial co-colonizer of CF patients with *C. albicans* infections was found to be *Staphylococcus aureus* (53.57%), with *P. aeruginosa* at 48.21%, *Staphylococcus maltophilia* at 16.07% and *Haemophilus influenza* at 5.97%. Other similar studies have also implicated *S. aureus* and *P. aeruginosa* as the most prevalent bacterial species isolated (Valenza et al., 2008; Williamson et al., 2011). Several studies addressed the correlation between *C. albicans* and *P. aeruginosa* in CF infection, with a recent study suggesting a significant correlation (Conrad and Bailey, 2015). Discrepancies may arise due to method of analysis and the population analyzed.

## Production and role of oxylipins during infection

Lipids have crucial cellular significance, forming membranes, as well as acting as cellular signals (reviewed by Tehlivets et al., 2007). The latter is of great importance in multicellular eukaryotic organisms. Although lipids as signaling molecules have been well studied in mammals and plants, their roles in fungi are not as well characterized.

**Figure 6:**
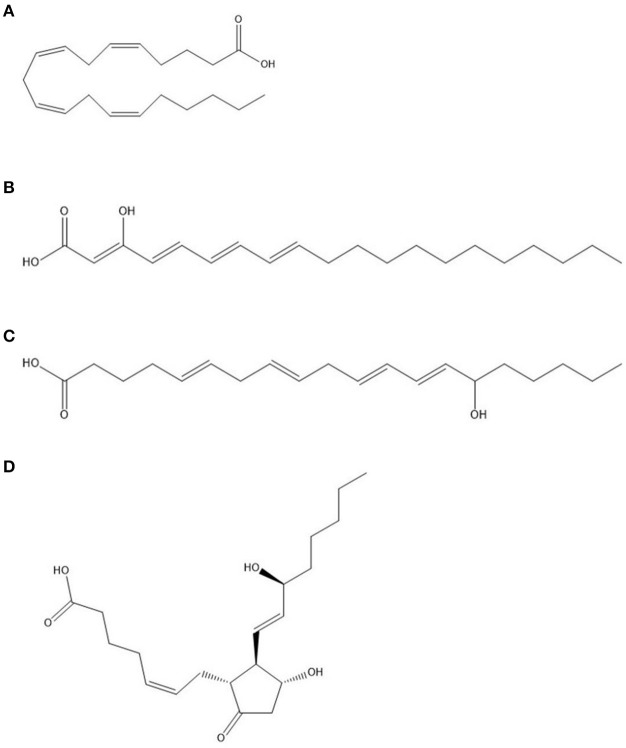
**Structures of (A) arachidonic acid (AA) at the top, followed by (B) 3-hydroxyeicosatetraenoic acid (3-HETE); (C) 15-HETE; (D) Prostaglandin E_2_ at the bottom**.

Arachidonic acid (AA) or 5,8,11,14-eicosatetraenoic acid (Figure 6A), a major constituent of the mammalian phospholipids, together with its wide range of metabolites (termed eicosanoids), have substantial roles as lipid signals (Chilton et al., 1996), including modulation of the innate immune response (Rodríguez et al., 2014). As the constituent of cellular membranes, it is predominantly found in the sn2 position of phospholipids with incorporation into inflammatory cells through CoA-dependant acyl transferases.

The metabolism of AA to form a plethora of lipid mediators involve various pathways in vertebrates, including the action of cyclooxygenases (COX), lipoxygenases (LOX), monooxygenases (CYP450), and non-enzymatic (NE) pathways. Cyclooxygenases, or prostaglandin endoperoxide synthases, are enzymes catalyzing the insertion of two oxygen atoms in AA (Marnett et al., 1999). In mammalian cells, two isoforms exist, namely, COX-1, which is constitutively expressed, and COX-2, which is inducible. The initial reaction of AA oxidation, mediated by COX, yields prostaglandin G_2_ (PGG_2_) (Rodríguez et al., 2014). Through peroxidase activity, PGG_2_ is reduced to prostaglandin H_2_ (PGH_2_), which serves as a precursor for various other immunomodulatory compounds, including prostaglandins as well as thromboxanes. For example, further action by Prostaglandin E synthase converts PGH_2_ to Prostaglandin E_2_ (PGE_2_) (Figure 6D).

Lipoxygenases are a large group of dioxygenases that catalyze oxygen insertion into polyunsaturated fatty acids in animals, plants as well as microorganisms (Kuhn and O'Donnell, 2006; Brodhun and Feussner, 2011). The reaction of oxygenation consists of various steps, starting with hydrogen abstraction, followed by radical rearrangement and the insertion of oxygen. In addition to oxygenation and hydroperoxidation, LOX also catalyze the synthesis of leukotrienes, lipoxins and hepoxilins through the combination of various enzymatic activities (Kuhn and O'Donnell, 2006; Dennis and Norris, 2015). Various isoforms of LOX exist with different stereospecificity and activities, for example 12/15-LOX catalyze oxygenation and hydroperoxidation of PUFAs at the 12 or 15 carbon position. Various studies suggest that the 12/15-LOX can be induced by interleukin-4 (IL4) and IL13, which are important in the Th2 response. Additionally, 12/15-LOX is inhibited by interferon ɤ. The low expression of 12/15-LOX has been linked to pro-inflammatory responses and has been implicated in airway functions. Prostaglandin E_2_ has also been argued to induce the expression of 12/15-LOX in neutrophils through cAMP elevation. Various products of LOX have also been implicated in anti-inflammatory responses in neutrophils. Many LOX products, including hydroperoxy-eicosatetraenoic acids (HPETEs) and hydroxy-eicosatetraenoic acids (HETEs) are intermediate products leading to the formation of lipoxins and leukotrienes (Dennis and Norris, 2015). The interaction of COX- and LOX-derived lipid mediators as well as the combination of these two pathways leads to the modulation of the inflammatory response (Dennis and Norris, 2015). The non-steroidal anti-inflammatory drug (NSAID), acetylsalicylic acid (ASA) (also known as aspirin), was shown to acetylate COX isozymes leading to the formation of 15(*R*)-HETE, which acts as LOX substrate for the formation of lipoxins (Serhan, 2002). These lipoxins are potent anti-inflammatory molecules, inhibiting neutrophil recruitment and leukotriene formation. In addition to these pathways, cytochrome P450s are also responsible for the formation of epoxyeicosatetraenoic acids (EETs) from AA, with concurrent modification to diHETEs, playing differential effects on the host.

The effects of eicosanoids on mammalian cells are dependent on the type of target tissue and the physiological state of these tissues (Dennis and Norris, 2015). Considerable research is being done to determine the eicosanoids that play a role in host protection against pathogens during infection as they can enhance the clearance of pathogens. For this review, the focus will fall on lipid mediators in terms of *C. albicans* and *P. aeruginosa* infection.

### Role of mammalian oxylipins during *Pseudomonas aeruginosa* infection

Several studies have addressed the effect of various invading pathogens on the production of PGE_2_ to gain a better understanding of the immunological aspects of infection. In mammalian cells, PGE_2_ is formed through the metabolism of AA by the action of cyclooxygenase (COX-1 or COX-2) to first form PGH_2_, followed by PGE synthases to form PGE_2_ (Murakami et al., 2003). Immune cells are the main source of PGE_2_, although this compound is also produced by various other cell types (Kalinski, 2012; Agard et al., 2013). It elicits a response in mammalian cells through activation of four receptors, designated EP1–EP4, with the effect dependent on the receptor activated.

*Pseudomonas aeruginosa* pulmonary infection is associated with an overproduction of PGE_2_ and concurrent decrease in phagocytosis by alveolar macrophages (Ballinger et al., 2006; Agard et al., 2013). This increase in PGE_2_ is due to the large amount of AA released during *P. aeruginosa* infection, mediated by ExoU, an intracellular phospholipase (König et al., 1996; Saliba et al., 2005; Sadikot et al., 2007; Agard et al., 2013). As such it plays a crucial role in initial infection and infiltration by *P. aeruginosa.* The absence of ExoU in *P. aeruginosa* was linked to diminished severity of infection and PGE_2_ production. The importance of PGE_2_ during *P. aeruginosa* infection was seen when a COX-2 inhibitor was employed, resulting in a decrease in severity of infection by this pathogen (Sadikot et al., 2007). Several other virulence factors also elicit changes in PGE_2_ levels. The QSM 3-oxo-HSL produced by *P. aeruginosa* was shown to induce COX-2 and, therefore, PGE_2_ production in human lung fibroblasts (Smith et al., 2002). Similarly, *P. aeruginosa* PYO and LPS increased the release of PGE_2_ in urothelial cells in a concentration dependent manner (McDermott et al., 2013).

### Role of *Pseudomonas aeruginosa* oxylipins during infection

A number of microorganisms including bacteria are able to form eicosanoids (Lamacka and Sajbidor, 1995). Although the presence of LOX in plants and animals has long been known, their presence in lower eukaryotes and prokaryotes has only recently been established, with *P. aeruginosa* one of the few bacteria with typical LOX genes. *Pseudomonas aeruginosa* has been found to possess a secretable 15-LOX, homologous to mammalian LOX, producing 15-HETE (Figure 6C) which is similar to host 15-HETE and elicits anti-inflammatory effects on the host, through acting as a substrate for lipoxin formation (Serhan, 2002; Vance et al., 2004). The formation of these lipoxins may alter the severity of infection through inhibiting neutrophil recruitment and generation of leukotrienes (Serhan, 2002). In addition to 15-HETE, *P. aeruginosa* is able to produce a range of products including dihydroxy unsaturated fatty acids such as 7,10-dihydroxy-8(*E*)-octadecenoic acid (DOD) (Hou, 2008). In addition, the production of prostaglandins and prostaglandin-analog compounds have been identified in *P. aeruginosa* (Lamacka and Sajbidor, 1995), however, the effect of these compounds during infection has not been addressed.

### Role of mammalian oxylipins during *Candida albicans* infection

The alteration of immune response is not unique to bacteria, but plays a significant role as a virulence factor during *C. albicans* infection. *Candida albicans* is able to metabolize AA, liberated from host cells by host as well as yeast-derived phospholipase A_2_ (Castro et al., 1994; Filler et al., 1994; Brash, 2001; Niewerth and Korting, 2001; Theiss et al., 2006; Parti et al., 2010). In addition, *C. albicans* can induce significant production of PGE_2_ by mammalian cells (Filler et al., 1994; Deva et al., 2001). *Candida albicans* mannans, forming part of the *C. albicans* cell wall, can directly induce PGE_2_ production by mammalian cells (Smeekens et al., 2010). In addition, the induction of the release of AA and the eicosanoid metabolites from alveolar macrophages has been shown to be partly regulated by mannose and β-glucan receptors interacting with fungal cell wall components and have also been shown to inhibit phagocytosis during *C. albicans* infection (Castro et al., 1994).

Eradication of infection by the immune system is highly dependent on the balance of Th1 and Th2 responses (Romani, 2000). The Th1 response is associated with the removal of pathogens through phagocytosis, in contrast to the hampering of this effect during the Th2 response. Prostaglandin E_2_ is able to modulate this balance and frequently promotes colonization of pathogens such as *C. albicans*, as well as causing tissue eosinophilia. Prostaglandin E_2_ production in response to *C. albicans* invasion also induces the protective Th-17 response in mammalian systems (Smeekens et al., 2010).

### Role of *Candida albicans* oxylipins during infection

AA can be used as a carbon source by *C. albicans* (Deva et al., 2000). The authors also identified a HETE produced by *C. albicans* from AA namely 3,18-diHETE, that is associated with hyphal forms and may play a role in adhesion during infection (Deva et al., 2000; Ells et al., 2012). Additionally, the authors showed that most AA is metabolized by non-mitochondrial pathways, concurrent with the speculation that fatty acids are metabolized by peroxisomal β-oxidation in yeasts. Acetylsalicylic acid-sensitive 3-HETE (Figure 6B), produced by *C. albicans* was also speculated to be linked to *C. albicans* morphogenesis (Deva et al., 2001). 3-HETE can also serve as substrate for COX-2 in mammalian cells to form 3-hydroxyeicosanoids due to the similarity to AA (Ciccoli et al., 2005). Further analysis of one of these metabolites, 3-OH-PGE_2_, indicated a similar or even more robust effect on mammalian cells eliciting a pro-inflammatory response.

The anti-inflammatory lipid Resolvin E_1_, which *Candida albicans* is able to produce from eicosapentaenoic acid, also plays a role in the interspecies signaling (Haas-Stapleton et al., 2007). This compound was shown to protect *C. albicans* against host immunity at low doses, however, at higher concentration, this protective effect is lost. This is thought to facilitate the commensal carriage of *C. albicans* as part of the human microbiome.

An important aspect of the determination of *C. albicans* virulence was the identification of a PGE_2_ cross reactive compound produced by *C. albicans* from exogenous AA (Noverr et al., 2001). This compound was later identified as PGE_2_, identical to the host product (Erb-Downward and Noverr, 2007). This was especially surprising as *C. albicans* does not possess homologs to COX present in mammalian cells responsible for the formation of PGE_2_. The involvement of various enzymes or homologous enzymes was speculated in the production of PGE_2_. A multicopper oxidase homolog (Fet3), fatty acid desaturase homolog (Ole2) and cytochrome P450s has been shown to be involved in the production of PGE_2_ by *C. albicans* (Erb-Downward and Noverr, 2007; Ells et al., 2011; Krause et al., 2015). Biofilm formation in *C. albicans* as well as germ tube formation has been shown to be enhanced by the addition of synthetic PGE_2_. In addition, various COX-inhibitors such as ASA drastically affect the formation of biofilms and germ tubes (Alem and Douglas, 2004). Interestingly, the simultaneous addition of PGE_2_ abolished the inhibitory effect of ASA on *C. albicans* biofilm formation. In a later study by the same researchers, a comparison of PGE_2_ production by *C. albicans* planktonic cells and biofilms showed a significant increase in PGE_2_ production of *C. albicans* biofilms (Alem and Douglas, 2005). In addition, various COX-inhibitors, namely ASA, diclofenac and etodolac, significantly decreased the production of PGE_2_ by *C. albicans* biofilms. These findings suggest a possible link between prostaglandin production and biofilm formation. Deva et al. (2001) reported significant decrease in yeast to hyphal transition by *C. albicans* with the addition of ASA. This morphological effect was attributed to the possible suppression of 3(R)-hydroxy-oxylipins.

**Figure 7:**
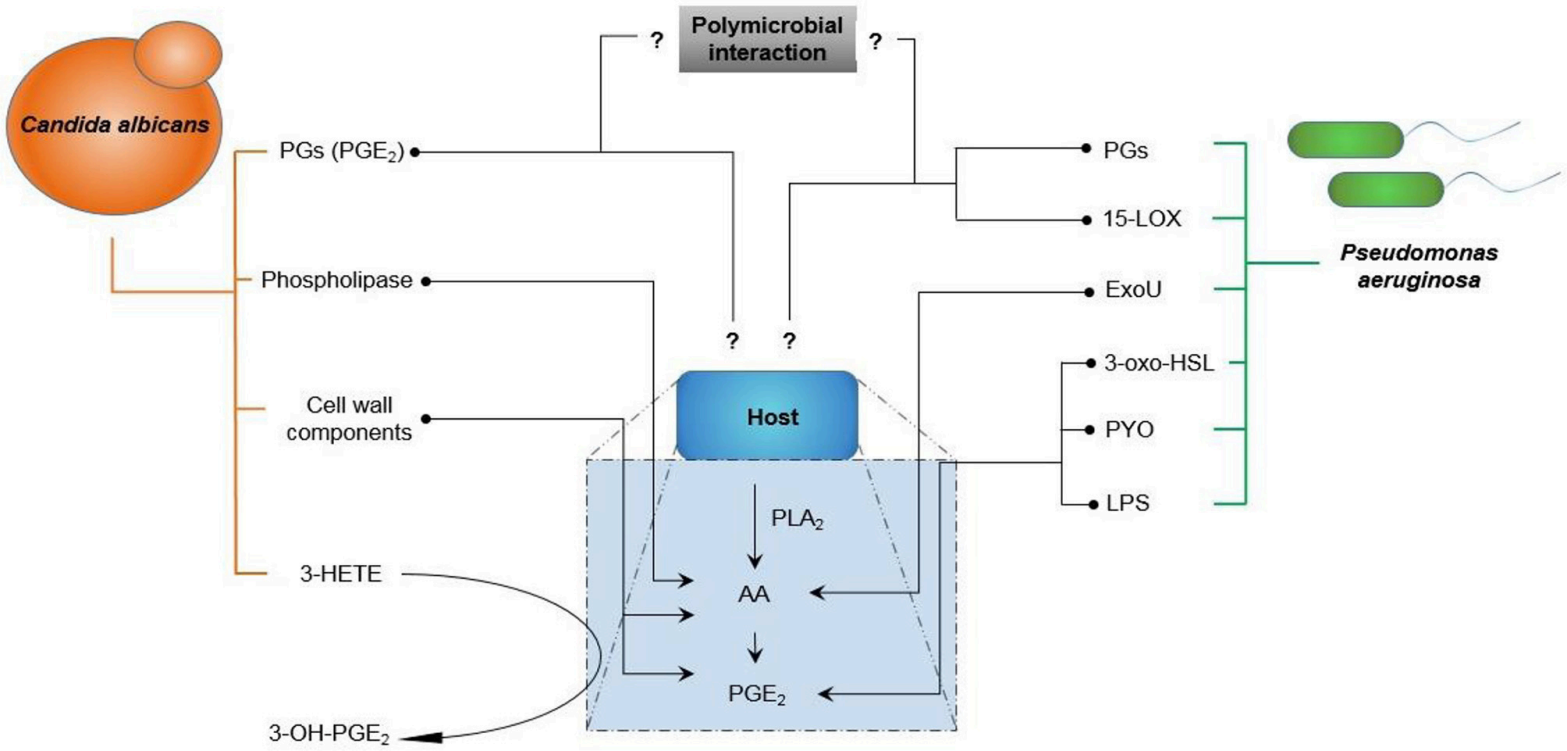
**Interaction of *Candida albicans* and *Pseudomonas aeruginosa* with host cells highlighting the production of eicosanoids**. Arrows indicate production/increase. Question marks indicate uncharacterized interactions (AA, Arachidonic acid; PLA_2_, Phospholipase A_2_; PGs, Prostaglandins; PGE_2_, Prostaglandin E_2_; ExoU, Exotoxin U; PYO, pyocyanin; LPS, lipopolysaccharide; 3-HETE, 3-hydroxyeicosatetraenoic acid; 3-oxo-HLS, 3-oxododecanoyl-L-homoserine lactone; 15-LOX, 15-Lipoxygenase).

Figure 7 indicates the interaction of *C. albicans* and *P. aeruginosa* with host cells with the focus on eicosanoid production. In the figure, arrows indicate the production or increase of an eicosanoid, whereas a question mark indicates an unknown effect or interaction.

Considering the information regarding the direct and indirect interaction of *C. albicans* and *P. aeruginosa*, as well as the information regarding the role of eicosanoids during single species infection, several questions can be asked relating to polymicrobial infection with *C. albicans* and *P. aeruginosa*.

### Role of oxylipins in polymicrobial infection

Although information is available regarding the eicosanoids produced, and their effect during single species infection, very little is known regarding the effect of microbially-produced eicosanoids on co-infecting pathogens, as well as on the host.

In a study by Peters and Noverr (2013), the role of eicosanoids during polymicrobial infection in another microbial interaction became evident. In this study, a murine model was used to elucidate the effect of polymicrobial interactions using *C. albicans* and *Staphylococcus aureus*. Through their work, it was evident that polymicrobial infections with these organisms resulted in a significant increase in morbidity and mortality during murine peritoneal infection, with this effect not seen with single species infection. In addition, disease progression and microbial load in infected mice was significantly higher, compared to monomicrobial infections. The authors also detected a significant increase in pro-inflammatory chemokines released, as well as recruitment of polymorphonucleocytes. To determine the effect of the pro-inflammatory response on infection dynamics, indomethacin was used. Indomethacin, a non-selective COX inhibitor, similar to ASA, caused a significant reduction in morbidity and mortality in polymicrobial infection, as well as a reduction in cytokine release. Indomethacin also caused a significant reduction in microbial load during co-infection. This effect was not seen with monomicrobial infection. *In vitro* experiments showed that this effect was not due to inhibition of growth of *C. albicans* and *S. aureus*. The authors also observed a significant increase in PGE_2_ release by the host cells in response to polymicrobial infection with indomethacin also causing a significant reduction in PGE_2_. The authors suggested that PGE_2_ may play a role in the non-protective pro-inflammatory response during polymicrobial infection, as PGE_2_ induces the release of several cytokines. To further determine the effect of PGE_2_ in co-infection, mice were co-infected with *C. albicans* and *S. aureus* and treated with indomethacin as well as PGE_2_. Under this circumstance, high morbidity and mortality was observed in the mice, although they were treated with indomethacin. In the absence of infection, with only the administration of indomethacin and PGE_2_, no mortality was observed. Further, the administration of PGE_2_ caused a significant increase in microbial burden during co-infection. During this study, the production of eicosanoids, such as PGE_2_, by *C. albicans* itself was not addressed. Interestingly, Krause et al. (2015) indicated that *C. albicans* PGE_2_ production significantly enhances *S. aureus* biofilm growth *in vitro*. The authors also found that co-incubation of *C. albicans* with *S. aureus* did not elicit an increase in PGE_2_ production by *C. albicans*. Nothing is known yet regarding the eicosanoid production of *C. albicans* and *P. aeruginosa* during co-infection.

## Conclusions

In addition to the ample evidence supporting the interaction of *C. albicans* and *P. aeruginosa*, not only with their host, but also with each other, it is evident that the interaction is multifaceted. Various virulence factors such as morphogenesis, hypermutabililty and secreted factors (including lipid mediators) affect and damage hosts to facilitate rapid and aggressive colonization and infection. Any disequilibrium in host defenses, such as in CF, immune disorders, and breaching of host barriers, is rapidly exploited by these opportunistic pathogens.

On their own, *C. albicans* and *P. aeruginosa* pose a risk to compromised hosts. Recent studies, however, illuminate that the interaction of these microorganisms are antagonistic with substantial damage caused to the host during the chemical war they play part in. Host immunity also plays a large role in damage to its own tissue due to radical generation by the innate immune response and various lipid mediators. This is even more evident in the work by Peters and Noverr (2013). It is also evident that various secreted factors of *C. albicans* (including farnesol and various hydrolytic enzymes) and *P. aeruginosa* (including AHL's, PQS, PYO and various peptides) form a large amount of radicals and can elicit oxidative damage to each other, as well as the host.

Although the role of lipid mediators between single pathogens and hosts has been studied, a gap in knowledge still exists regarding the effect of lipid mediators in polymicrobial infection of *C. albicans* and *P. aeruginosa*. Polymicrobial infection by *C. albicans* and *S. aureus*, however, highlight the importance of lipid mediators, such as PGE_2_, in infection by multiple microbes. Due to the fact that *C. albicans* and *P. aeruginosa* are both able to produce significant amounts of prostaglandins and other eicosanoids from exogenous AA, this could affect the dynamics of this co-infection as well as host survival during infection. This warrants investigation to fully understand the interaction of *C. albicans* and *P. aeruginosa* in terms of eicosanoids, as well as the effect of these eicosanoids in the host during co-infection.

## Author contributions

RF compiled the information, co-wrote the manuscript and approved the final version submitted. RE, CW, OS, JA provided scholarly input in placing the literature into context, edited the manuscript and approved the final version submitted. CP provided scholarly input in placing the literature into context, co-wrote the manuscript and approved the final version submitted

## Funding

The financial assistance of the National Research Foundation (NRF) (Grant: CPRR 93485) toward this research is hereby acknowledged. Opinions expressed and conclusions arrived at, are those of the author and are not necessarily to be attributed to the NRF.

### Conflict of interest statement

The authors declare that the research was conducted in the absence of any commercial or financial relationships that could be construed as a potential conflict of interest. The reviewer AH and handling Editor declared a common affiliation and the handling Editor states that the process nevertheless met the standards of a fair and objective review.
